# The Relation of Hepcidin to Iron Disorders, Inflammation and Hemoglobin in Chronic Kidney Disease

**DOI:** 10.1371/journal.pone.0099781

**Published:** 2014-06-30

**Authors:** Lucile Mercadel, Marie Metzger, Jean Philippe Haymann, Eric Thervet, Jean-Jacques Boffa, Martin Flamant, François Vrtovsnik, Pascal Houillier, Marc Froissart, Bénédicte Stengel, the NephroTest Study Group, François Vrtovsnik, Eric Daugas, Martin Flamant, Emmanuelle Vidal-Petiot, Christian Jacquot, Alexandre Karras, Eric Thervet, Christian d’Auzac, P. Houillier, M. Courbebaisse, D. Eladari et G. Maruani, Jean-Jacques Boffa, Pierre Ronco, H. Fessi, Eric Rondeau, Emmanuel Letavernier,, Jean Philippe Haymann, P. Urena-Torres

**Affiliations:** Bichat Hospital; Bichat Hospital; Bichat Hospital; Bichat Hospital; European Georges Pompidou Hospital; European Georges Pompidou Hospital; European Georges Pompidou Hospital; European Georges Pompidou Hospital; European Georges Pompidou Hospital; European Georges Pompidou Hospital; European Georges Pompidou Hospital; Tenon Hospital; Tenon Hospital; Tenon Hospital; Tenon Hospital; Tenon Hospital; Tenon Hospital; clinique du Landy, Saint-Ouen; 1 Inserm, Centre for research in Epidemiology and Population Health, U1018, Epidemiology of Diabetes, Obesity, and Kidney Diseases Team, Villejuif, France; 2 Department of Nephrology, Hôpital Pitié-Salpêtrière, Assistance Publique-Hôpitaux de Paris, Paris, France; 3 Université Paris Sud 11, U1018, Villejuif, France; 4 Department of Physiology and Nephrology, Hôpital Tenon, Assistance Publique-Hôpitaux de Paris, Université Pierre et Marie Curie, Inserm U702, Paris, France; 5 Department of Nephrology, Hôpital Européen G Pompidou, Assistance Publique-hôpitaux de Paris, Paris, France; 6 Department of Nephrology, Hôpital Tenon, Assistance Publique-hôpitaux de Paris, université Pierre et Marie Curie, Paris, France; 7 Department of Physiology, Hôpital Bichat, Assistance Publique-hôpitaux de Paris, Paris, France; 8 Inserm U699, Paris, France; 9 Department of Nephrology, Hôpital Bichat, Assistance Publique-hôpitaux de Paris, Université Pierre et Marie Curie, Paris, France; 10 Department of Physiology, Hôpital Européen G Pompidou, Assistance Publique-hôpitaux de Paris, Université Paris Descartes, Paris, France; University Medical Center Utrecht, Netherlands

## Abstract

The metabolism of hepcidin is profoundly modified in chronic kidney disease (CKD). We investigated its relation to iron disorders, inflammation and hemoglobin (Hb) level in 199 non-dialyzed, non-transplanted patients with CKD stages 1–5. All had their glomerular filtration rate measured by ^51^Cr-EDTA renal clearance (mGFR), as well as measurements of iron markers including hepcidin and of erythropoietin (EPO). Hepcidin varied from 0.2 to 193 ng/mL. The median increased from 23.3 ng/mL [8.8–28.7] to 36.1 ng/mL [14.1–92.3] when mGFR decreased from ≥60 to <15 mL/min/1.73 m^2^ (p = 0.02). Patients with absolute iron deficiency (transferrin saturation (TSAT) <20% and ferritin <40 ng/mL) had the lowest hepcidin levels (5.0 ng/mL [0.7–11.7]), and those with a normal iron profile (TSAT ≥20% and ferritin ≥40), the highest (34.5 ng/mL [23.7–51.6]). In multivariate analysis, absolute iron deficiency was associated with lower hepcidin values, and inflammation combined with a normal or functional iron profile with higher values, independent of other determinants of hepcidin concentration, including EPO, mGFR, and albuminemia. The hepcidin level, although it rose overall when mGFR declined, collapsed in patients with absolute iron deficiency. There was a significant interaction with iron status in the association between Hb and hepcidin. Except in absolute iron deficiency, hepcidin’s negative association with Hb level indicates that it is not down-regulated in CKD anemia.

## Introduction

Hepcidin is a new iron marker, discovered in 2001 and studied especially in hemochromatosis. The first investigations concerned prohepcidin, an inactive precursor. Prohepcidin has no impact on iron metabolism in either healthy individuals [1] or patients with chronic kidney disease (CKD) [2], [3]. The involvement of hepcidin in iron disorders was clarified with the development of methods to quantify hepcidin-25 [2], [4]. Hepcidin has been evaluated in absolute iron deficiency, where its concentration is low, and in patients with chronic disease-related anemia [5]. The subsequent development of more specific assays for hepcidin-25 has led to a redefinition of normal values from around 50 ng/mL for the first assays to less than 10 ng/mL for those recently validated by the FDA [6]. Except in hemochromatosis, however, hepcidin has not yet proved its usefulness in clinical practice.

Hepcidin involvement in CKD anemia has been explored in both dialysis and non-dialysis CKD patients: both CKD-related inflammation and lower hepcidin clearance tend to increase hepcidin concentration in these patients [7], [8], [9]. The specificity of hepcidin for the diagnosis of absolute iron deficiency may thus be limited in this group. The hepcidin level does not seem to improve the prediction of erythropoietin response [10], [11]. Furthermore, its close relation with ferritin raises questions about its performance in the diagnosis of iron disturbances [12]. In hemodialysis patients, its performance is no better than that of traditional iron markers [2], [12]. Nonetheless, a better understanding of the determinants of hepcidin levels is essential to assess its usefulness in CKD anemia. We therefore studied the determinants of hepcidin in non-dialysis CKD patients, particularly its relation to iron disorders, inflammation, and glomerular filtration rate measured (mGFR) by a reference method. We also tested the hypothesis that hepcidin and hemoglobin (Hb) levels are related, independently of other Hb determinants.

## Methods

### Population

The NephroTest study is a prospective hospital-based cohort that has enrolled patients with any diagnosis of CKD stages 1 through 5, recruited from three nephrology departments. Patients younger than 18 years, dialysis patient or with a kidney transplant, and pregnant women were excluded. Between January 2000 and January 2012, NephroTest included 1095 patients after they had provided written informed consent. The NephroTest study design was approved by the relevant ethics committee (Direction Générale pour la Recherche et l’Information, Comité Consultatif sur le Traitement de l’Information en matière de Recherche dans le domaine de la Santé MG/CP09.503) and adheres to the Declaration of Helsinki. Patients not receiving EPO and intravenous iron were included in this analysis if a plasma sample was available for them. More precisely, 114 men were drawn from the NEPHROTEST cohort samples from 2000 to 2004 and all women of the same period were included (n = 48). Additionally, 37 women with plasma samples available from the 2006 to 2008 period were added to obtain a study population that was balanced with respect to sex. Hepcidin was measured for these 199 patients. They were similar to the overall study population with respect to age and mGFR distribution (Appendix 1). Biopsy-proven nephropathy was identified in 21% of all patients, but fewer than 10% of those with diabetes. Clinical criteria based on a history of urinary albumin >300 mg/g creatinine and of other microangiopathy damage (retinopathy or neuropathy) were used to classify diabetes patients who had not had renal biopsies with diabetic glomerular nephropathy. Other diabetes patients were classified with another nephropathy type, most probably vascular.

### Laboratory measurements

Patients undergo an extensive annual check-up, including a measurement of their GFR (mGFR) by *Cr-EDTA, in the hospitals’ physiology departments [13]. Serum iron (DxC800 Beckman-Coulter, ferrozine, emitted light 560 nm), ferritin (BN-Siemens, N-latex ferritin immunonephelometry), and transferrin (BN-Siemens, N Antiserum antitransferrin immunonephelometry) were measured. TIBC (total iron binding capacity, µmol/L) was calculated as 25×transferrin (g/L) and TSAT (%) as serum iron×100/TIBC. Firstly, we used Lipschitz’s iron index [13] to define iron status based on TSAT and ferritin together as follows: normal, TSAT ≥20%; absolute iron deficiency, TSAT <20% and ferritin <40 ng/mL; functional iron deficiency, TSAT <20% and ferritin ≥40 ng/mL. The 40 ng/mL threshold value was chosen for ferritin because this threshold is usually recommended to diagnose absolute iron deficiency, in contrast with the KDIGO threshold of 100 ng/mL used to define the need for iron supply in patients with non-dialysis chronic kidney disease. Secondarily, we used a combined iron marker containing 4 classes, built like the Lipschitz iron index but separating TSAT ≥20% and ferritin ≥40 ng/mL from TSAT ≥20% and ferritin <40 ng/mL. Endogenous EPO levels were determined in serum (100 µL) with the Quantitine IVD Epo double-antibody sandwich ELISA method from R&D Systems (Minneapolis, MN), as reported elsewhere [14]. EPO measurements were missing for 38 patients (37 women and 1 men).

The assay for the quantification of hepcidin in human serum was developed at Amgen, Thousand Oaks, CA, and is described in appendix 2. Briefly, this immunoassay is based on hepcidin capture by an anti-human hepcidin monoclonal antibody, followed by electrochemiluminescent detection of the complex. The normal reference level of hepcidin in healthy volunteers was <10 ng/mL. The lower limit of detection was 0.1 ng/mL.

### Statistical analyses

We studied crude and mGFR-adjusted relations of hepcidin with age, gender, ethnicity, diabetes and diabetic nephropathy, body mass index (BMI), mGFR, the urinary protein to creatinine ratio (PCR), albuminemia, C-reactive protein (CRP), oral iron treatment, erythropoietin, ferritin, TSAT, TIBC, and the combined iron marker; we used ANOVA to compare categorial variables and Pearson’s correlations for quantitative variables. The hepcidin determinants were then analysed by multivariate regression analysis that included age, gender, center, albumin, BMI, CRP, mGFR, EPO, oral iron therapy, and the combined iron marker. Finally, we tested the association of Hb levels with the hepcidin concentration treated continuously after adjustment for other Hb determinants. These included the combined iron marker (or ferritin), gender, diabetes, BMI, mGFR, oral iron treatment, albuminemia, CRP, and angiotensin converting enzyme inhibitors/angiotensin receptor blockers. Potential interactions between hepcidin and the combined iron marker (or ferritin) in the relation with Hb were also tested. In all analyses, hepcidin was transformed by its square root to meet the criterion of a normal parameter.

For covariates with less than 3% missing observations (albumin and CRP), the median value was imputed in the multivariate analysis. A missing data category was created for the combined iron marker and EPO. Statistical analyses were performed with SAS 9.2 (SAS Institute Inc., Cary, NC, USA) and R 2.15 (R Foundation for Statistical Computing, Vienna, Austria, 2012).

## Results

### Patients’ characteristics

Patients’ characteristics are shown in Table 1. Hepcidin ranged from 0.2 to 193 ng/mL with a median value of 27.9 ng/mL [IQR 16.5–45.4]. According to Lipschitz’s iron index [15], 72.4% had normal iron profiles, 6.3% absolute iron deficiency and 21.4% functional iron deficiency. This distribution was similar to that observed in the entire cohort (71.3%, 6.2%, and 18.6%). The median mGFR for each class of the combined iron marker was 34.7 mL/min/1.73 m^2^ [IQR 24.0–48.3], 37.2 mL/min/1.73 m^2^ [24.8–56.0], and 36.0 mL/min/1.73 m^2^ [26.3–49.5].

**Table 1:** Patient characteristics.

	Mean ± SD, median (IQR) or %(N)
Men	57.3 (114)
Age, years	58.4±14.8
African origin	5.8 (11)
Body mass index, kg/m^2^	25.5±5.0
Systolic/Diastolic Blood Pressure, mmHg	138±20/76±11
Diabetes	
No	76.4 (152)
Yes, with diabetic nephropathy	15.1 (30)
Yes, with other nephropathy type	8.5 (17)
History of cardiovascular disease	12.6 (25)
mGFR, ml/min/1.73 m^2^	35.3 (24.2–49.3)
<15	7.0 (14)
15–30	28.1 (56)
30–45	33.7 (67)
45–60	18.1 (36)
>60	13.1 (26)
eGFR MDRD, ml/min/1.73 m^2^	33.3 (22.6–45.9)
eGFR CKD-EPI, ml/min/1.73 m^2^	34.2 (23.2–48.4)
PCR, mg/mmol	28.9 (15.3–115.1)
Serum albumin, g/L	39.53±5.18
C-reactive protein >8 mg/L	11.0 (21)
Hb, g/dL	12.32±1.47
WHO Anemia	54.8 (109)
Serum Iron, µmol/L	14.06±5.16
Total iron binding capacity (TIBC), µmol/L	56.5±10.9
Transferrin saturation (TSAT), %	25.47±9.83
Ferritin, ug/L	110 (63–201)
Folates, ng/L	6.8 (5.0–9.5)
Vitamin B12, pg/mL	418 (291–558)
ACEi or ARB %	68.3 (136)
Oral iron therapy	11.1 (22)
Lipschitz’s iron index	
* TSAT ≥20%*	72.4 (139)
* TSAT <20% and ferritin <40*	6.3 (12)
* TSAT <20% and ferritin ≥40*	21.4 (41)

PCR: urinary protein to creatinine ratio; ACEi/ARBs: angiotensin converting enzyme inhibitors/angiotensin receptor blockers. WHO anemia: Hb <13 g/dL for men and <12 g/dL for women.

### Factors associated with hepcidin levels

**Figure 1:**
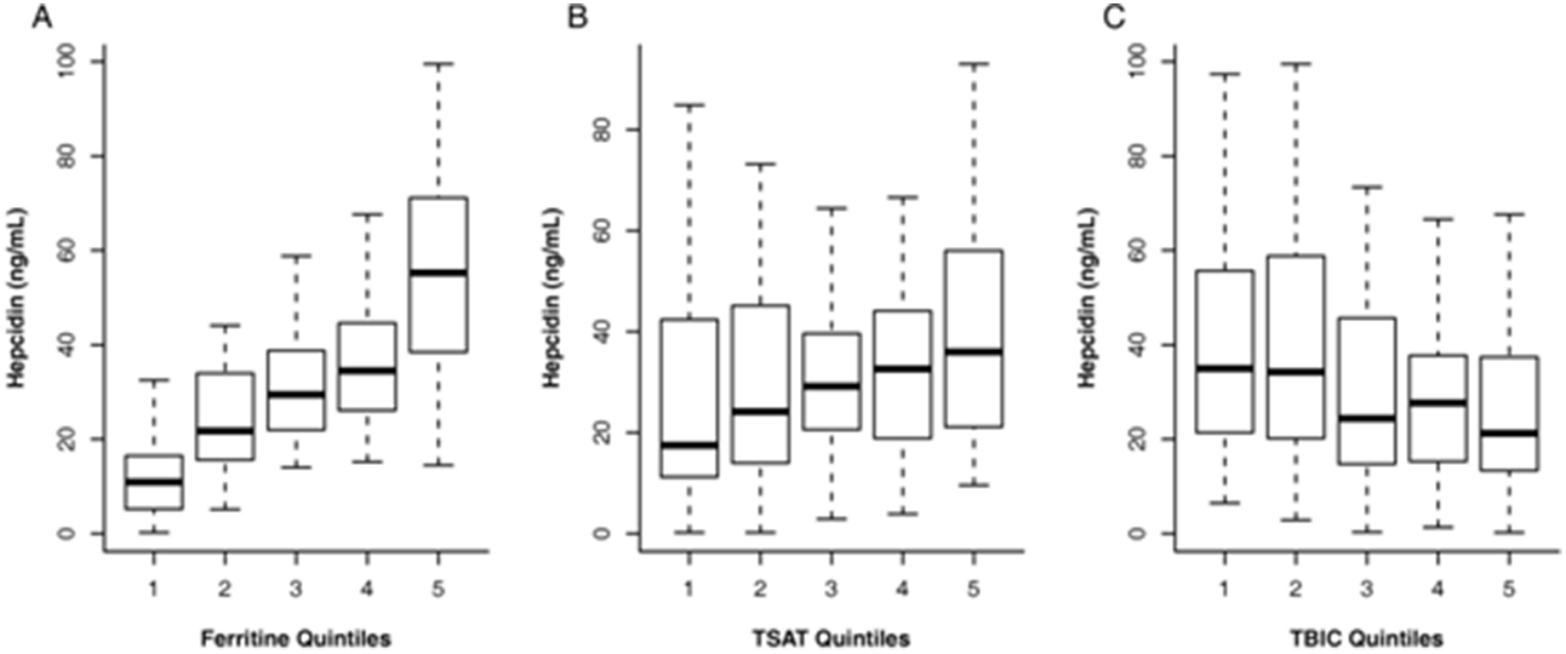
Hepcidin concentration according to quintiles of ferritin (A), transferrin saturation (B) and total iron-binding capacity (C).

Women younger than 55 years had lower hepcidin values than either men or older women (Table 2). Hepcidin levels increased from 23.3 ng/mL IQR [8.8–28.7] to 36.1 ng/mL IQR [14.1–92.3] when mGFR declined from >60 mL/min/1.73 m^2^ to <15 mL/min/1.73 m^2^. Independent of mGFR, higher levels of body mass index, albuminemia, C-reactive protein (CRP), oral iron therapy and lower levels of proteinuria and EPO were significantly associated with higher hepcidin concentrations (Tables 2 and 3). Hepcidin levels were strongly related to all iron markers and particularly to ferritin (Table 3 and Figure 1). The hepcidin level varied in the different combined classes of TSAT and ferritin (Table 4). It was six times lower in patients with absolute iron deficiency and three times in those with low ferritin alone than in those with normal iron status. The multivariate analysis showed that oral iron therapy, albuminemia, EPO levels, BMI ≥ 30 kg/m^2^ and mGFR all remained significantly associated with hepcidin levels. The four-class iron index produced the model with the best fit (Table 5). There was an interaction between iron status profile and CRP in their relation with hepcidin (p = 0.01). Patients with inflammation and either a functional or normal iron profile had higher hepcidin levels than their counterparts without inflammation (Table 5). Patients with ferritin <40 ng/mL had an hepcidin collapse.

**Table 2:** Hepcidin level according to categorial factors.

		N	Hepcidin (ng/mL)Median (IQR)	p-value	mGFR adjustedp-value
Age×Gender	Men	114	27.7 (19.0–43.1)	0.009	0.003
	Women <55 yrs	35	15.6 (8.8–40.2)		
	Women ≥55 yrs	50	34.6 (21.3–57.4)		
Origin	African	11	34.5 (8.0–63.9)	0.6	0.5
	Caucasian or other	180	27.7 (16.6–43.6)		
	missing	8	47.6 (16.2–66.0)		
Diabetes	No	152	28.8 (17.7–46.1)	0.8	0.8
	Yes with diabetic nephropathy	30	26.2 (12.5–45.2)		
	Yes with othernephropathy type	17	21.4 (16.3–42.4)		
Oral Iron Therapy	No	177	27.1 (16.0–42.3)	0.005	0.01
	Yes	22	46.5 (19.0–75.9)		
ACEI/ARBs	No	63	26.5 (16.0–48.3)	0.8	0.8
	Yes	136	29.3 (17.2–44.7)		
CRP	≤8 mg/L	170	26.7 (16.4–40.4)	0.0002	0.0005
	>8	21	56.4 (26.1–73.2)		
	missing	8	23.8 (7.7–87.7)		

CRP: C reactive protein; ACE/ARBs: angiotensin converting enzyme inhibitors/angiotensin receptor blockers.

**Table 3:** Crude and mGFR-adjusted Pearson’s correlations of square root-transformed hepcidin with quantitative factors.

	Crude correlation		mGFR-adjusted partial correlation	
	r	p-value	r	p-value
mGFR	−0.31	<.0001	-	-
BMI	0.21	0.003	0.16	0.03
LOG PCR	0.01	0.9	−0.18	0.01
Albuminemia	0.04	0.6	0.17	0.02
Free iron	0.05	0.5	0.18	0.01
TIBC	−0.32	<.0001	−0.22	0.002
TSAT	0.15	0.04	0.24	0.0008
LOG Ferritin	0.62	<.0001	0.71	<.0001
LOG EPO	−0.20	0.01	−0.18	0.03

mGFR: measured glomerular filtration rate; BMI: body mass index; PCR: urinary protein to creatinine ratio; CRP: C-reactive protein; EPO: erythropoietin; TIBC: total iron binding capacity; TSAT: transferrin saturation.

**Table 4:** Hepcidin levels (ng/mL) and percentage of patients with low hepcidin values according to ferritin and transferrin saturation (TSAT) levels.

		Ferritin		
		≥40 ng/mL	<40 ng/mL	Total
**TSAT**	≥20%	34.5 (23.7–51.6)^2^10% (12) N = 123	10.3 (6.9–14.1)^2^80% (12) N = 15	31.2 (20.9–45.7)^1^17% (24) N = 138
	<20%	22.6 (16.3–43.1)^1^^,^ 229% (12) N = 41	5.0 (0.7–11.7)^1^^,^ 292% (11) N = 12	19.2 (12.2–40.3)43% (23) N = 53
	Total	32.1 (21.35–51.3)15% (24) N = 164	9.6 (3.8–12.7)85%(23) N = 27	

Median (Interquartile range).

% (N) of patients with hepcidin values below a threshold value of hepcidin defined as the 10^th^ percentile of patients with normal iron profile (16.7 ng/mL).

1 Three classes of Lipschitz’s iron index.

2 Four classes iron index.

**Table 5:** Multivariate analyses of hepcidin levels according to different definitions of iron status.

	N	BIC/AIC	√hepcidinβ±sd	p-value
**Lipschitz’s iron index** 1 **^,^** 2		877/821		
TSAT ≥20%	139		ref	
TSAT <20% and Ferritin <40 µg/L	12		−2.71±0.55	<.0001
TSAT <20% and Ferritin ≥40 µg/L	41		−0.32±0.32	0.3
Missing	7		−0.04±0.71	0.9
**Four classes iron index** 1 **^,^** 2		848/788		
TSAT ≥20% and Ferritin ≥40 µg/L	123		ref	
TSAT ≥20% and Ferritin <40 µg/L	15		−2.84±0.46	<.0001
TSAT <20% and Ferritin <40 µg/L	12		−3.25±0.51	<.0001
TSAT <20% and Ferritin ≥40 µg/L	41		−0.65±0.30	0.03
Missing	8		−0.16±0.61	0.8
**Four classes iron index according to CRP levels** 1		848/785		
TSAT ≥20%, Ferritin ≥40 µg/L				
CRP ≤8 ng/mL	115		ref	
CRP >8 ng/mL	8		1.87±0.61	0.002
TSAT ≥20% and Ferritin <40 µg/L	15		−2.54±0.46	<.0001
TSAT <20% and Ferritin <40 µg/L	12		−3.09±0.51	<.0001
TSAT <20%, Ferritin ≥40 µg/L				
CRP ≤8 ng/mL	32		−0.90±0.32	0.005
CRP >8 ng/mL	9		2.33±0.57	<.0001
Missing	8		0.06±0.60	0.9

*Abbreviations: TSAT, Transferrin saturation; CRP, C-reactive protein; BIC, Bayesian Information Criterion; AIC, Akaike information Criterion; Ref, reference class.*

**β±sd** Regression coefficients for the different iron indexes in the linear regression models of hepcidin values (square-root transformed).

1 Models were adjusted for measured glomerular filtration rate, gender, age, body mass index, albuminemia, erythropoietin, oral iron and centre.

2 Models for Lipschitz’s iron index and the four-class iron index were also adjusted for C-reactive protein (CRP) in two classes (≤8 vs >8 ng/mL).

### Relation between Hb and hepcidin levels

There was a significant interaction with the four-class iron index in the relation between hepcidin and Hb (p = 0.003). Hb levels tended to decrease as hepcidin levels increased when TSAT <20% and ferritin >40 µg/L (Hb = −0.14±0.08, p = 0.06), but the relation was reversed in patients with absolute iron deficiency, defined by TSAT <20% and ferritin <40 µg/L (Hb = 0.30±0.14, p = 0.04). These relations were not modified when the four-class iron index was combined with inflammation (data not shown). Using ferritin instead of the combined iron marker produced similar results for the relation between hepcidin and Hb, with significant interaction between tertiles of ferritin and hepcidin (p<0.001). Hb levels decreased significantly with increasing hepcidin at intermediate (74–167 ng/mL, Hb = −0.22±0.09, p = 0.02) and high ferritin values (>167 ng/mL, Hb = −0.20±0.08, p = 0.01), and increased with hepcidin at low ferritin values (<74 ng/mL, Hb = 0.15±0.07, p = 0.04). Further adjustment for the EPO level did not change the association of Hb and hepcidin (data not shown).

## Discussion

This study identified several factors other than mGFR that may determine hepcidin levels in CKD patients. The most original finding is our showing that even though hepcidin levels generally increase as mGFR declines, CKD patients with absolute iron deficiency still experienced a profound hepcidin collapse, in contrast to those with other iron profiles. We also confirmed the observation of an interaction between iron status and hepcidin in their relation to Hb levels. These findings are especially noteworthy because they are based on a patient population carefully phenotyped for mGFR and a specific and accurate hepcidin-25 assay with a low limit of detection.

Hepcidin is influenced by four regulation processes: hypoxia [16], erythropoiesis, inflammation, and iron status. We confirmed the main determinant factors of hepcidin seen in the general population [17] and in the hemodialysis population [18], [19], [20]. EPO deficiency and the chronic inflammation observed in CKD patients play a role in the hepcidin increase. BMI was a determinant of hepcidin, independently of inflammation. Some hepcidin secretion is located in and directly related to the adipose tissue [21]. Finally, iron status influences hepcidin levels. A low iron store down-regulates hepcidin, in a relation modulated by transferrin [22], [23]. Our 3-marker iron index with five classes produced similar results in this analysis (data not shown) [24]. In our cohort, the combined iron marker we used to classify the different iron profiles sheds light on the persistent process of hepcidin down-regulation in absolute iron deficiency at the same time that the patients with all other iron profiles had increasing hepcidin levels associated with mGFR decline. This persistent down-regulation has also been shown in animal models of chronic disease anemia [5]. The down-regulation process remains effective in and specific to absolute iron deficiency in CKD patients. The possibility of defining a hepcidin threshold value for this diagnosis requires further validation in CKD cohorts.

The influence of mGFR on hepcidin has been interpreted in different ways in recent articles reporting the role of the hepcidin assay. Hepcidin measurement is based on either mass spectrometry or antibody-based hepcidin detection. Competitive radioimmunoassays and ELISA tests have been developed for the antibody-based methods. Several studies have reported that hepcidin increases as mGFR declines [4], [17], [25]. The hepcidin measurements in these articles, however, were performed with a radioimmunoassay that might have cross-reacted with the isoform hepcidin-20 for about 10% of the total measured value of hepcidin-25. Further contestation of the relation between mGFR and hepcidin has been based on suspicion that this relation is restricted to hepcidin-20, which is inactive [26]. A large cohort study of 505 CKD patients that used a highly specific liquid chromatography tandem mass-spectrometry method found that estimated GFR was associated with hepcidin levels in the univariate but not the multivariate analysis [27]. Finally, from a physiological point of view, the isoform hepcidin-25, the active form, is a small peptide, freely filtered by the glomerulus and highly reabsorbed by the proximal tubules with an excretion fraction around 8%; it shares the renal clearance characteristics of β2 microglobulin [28], [29]. A major strength of our study is the use of ^51^Cr-EDTA renal clearance to determine GFR, which reduced measurement errors and improved our ability to understand the relation between renal function and hepcidin level. It is likely that levels of the serum isoform hepcidin-25 increase with declining renal function, but the relation between mGFR and hepcidin may have been disrupted by patients with absolute iron deficiency because of their down-regulated hepcidin level. Besides the difference in methods, differences in study populations could have influenced the relation between hepcidin and mGFR in some studies.

Anemia in CKD cohorts has been associated with elevated hepcidin levels. This association reflects the ratios of the prevalence rates of the different iron disturbances: absolute ID, which depresses hepcidin, is relatively rare in CKD cohorts such as ours (6.3%). Functional iron deficiency is more frequent and is associated with an elevated hepcidin level. Our functional iron profile group had elevated hepcidin levels, similar to those of the normal iron profile group. Combining a functional iron deficiency and overt inflammation further increased hepcidin in our study. The short half-life of hepcidin (22 hours), its high intra-patient variability, and the cross-sectional design of our study might have weakened the link between functional iron deficiency and a hepcidin increase. Recently in a mouse model, the hepcidin increase has been proved to be limited to the early phase of the inflammation process [30].

We confirmed that the relation between Hb and hepcidin varies according to iron status. In the study by Uehata et al [27], hepcidin was not related to Hb in the low ferritin group (<91 ng/mL) and was negatively related to it in the high ferritin group. The low ferritin group probably had absolute iron deficiency, with a very low hepcidin level of 7 ng/mL (IQR 2.3–17.3 ng/mL, with liquid chromatography mass spectrometry). We showed that in the absolute iron deficiency group, Hb decreased with hepcidin. On the contrary, in patients with functional iron deficiency, anemia was related to an increased hepcidin level. The hepcidin level thus provides information about Hb above and beyond that contained in the iron profiles. Elevated hepcidin can predict poor gut absorption of iron and ineffective oral iron treatment. In vitro studies suggest that hepcidin has a direct effect on erythropoiesis by inhibiting erythroid colony formation when erythropoietin is low and by impairing red blood cell survival. To date, however, no study has found that hepcidin has any predictive value for response to either IV iron or ESA.

The limitations of our study are linked mainly to its cross-sectional design, its sample size, and the measurement method. The cross-sectional design makes it difficult to infer causality between the hepcidin level and the risk of anemia. In terms of sample size among non-dialysis CKD cohorts, our study is the second largest examining hepcidin levels, after that of Uehata et al [27]. The immunochemical method that we used measured hepcidin levels in patients with absolute iron deficiency similar to those in the study of Uehata et al, which used mass spectrometry. The antibody-based hepcidin assays may diagnose and discriminate absolute iron deficiency quite well. On the contrary, when hepcidin values are elevated, the lower specificity for hepcidin-25 of the immunochemical test we used might have impaired its power to discriminate patients with normal iron profile from those with functional iron deficiency profile.

In conclusion, this study primarily shows the importance of considering iron profiles in improving our understanding of the changes of the hepcidin concentration as renal function declines as well as its association with CKD anemia. While absolute iron deficiency is associated with a profound hepcidin collapse independently of the GFR decline, hepcidin is elevated in all other CKD patients and thus impairs the oral iron absorption in these patients. These findings have prompted research on drug development focusing on a hepcidin antagonist in CKD and possibly other chronic disease anemia with functional iron deficiency, although the possible side effects of these drugs may limit their potential.
