# Correction: The Relation of Hepcidin to Iron Disorders, Inflammation and Hemoglobin in Chronic Kidney Disease

**DOI:** 10.1371/journal.pone.0123145

**Published:** 2015-03-26

**Authors:** The PLOS ONE Staff

The first author’s name is spelled incorrectly. The correct name is: Lucile Mercadal. The correct citation is: Mercadal L, Metzger M, Haymann JP, Thervet E, Boffa J-J, Flamant M, et al. (2014) The Relation of Hepcidin to Iron Disorders, Inflammation and Hemoglobin in Chronic Kidney Disease. PLoS ONE 9(6): e99781. doi:10.1371/journal.pone.0099781
